# Vitamin D: Deficiency, Sufficiency and Toxicity

**DOI:** 10.3390/nu5093605

**Published:** 2013-09-13

**Authors:** Fahad Alshahrani, Naji Aljohani

**Affiliations:** 1 Department of Medicine, King Abdulaziz Medical City, Riyadh 14611, Saudi Arabia; E-Mail: fahad_alshahrani@yahoo.com; 2 Specialized Diabetes and Endocrine Center, King Fahad Medical City, Riyadh 59046, Saudi Arabia; E-Mail: najijohani@gmail.com; 3 Faculty of Medicine, King Saud bin Abdulaziz University for Health Sciences, Riyadh 22490, Saudi Arabia; 4 Prince Mutaib Chair for Biomarkers of Osteoporosis, College of Science, King Saud University, Riyadh 11451, Saudi Arabia

**Keywords:** vitamin D, vitamin D deficiency, vitamin D toxicity

## Abstract

The plethora of vitamin D studies over the recent years highlight the pleomorphic effects of vitamin D outside its conventional role in calcium and bone homeostasis. Vitamin D deficiency, though common and known, still faces several challenges among the medical community in terms of proper diagnosis and correction. In this review, the different levels of vitamin D and its clinical implications are highlighted. Recommendations and consensuses for the appropriate dose and duration for each vitamin D status are also emphasized.

## 1. Introduction

Vitamin D plays an essential role in the regulation of metabolism, calcium and phosphorus absorption of bone health. However, the effects of vitamin D are not limited to mineral homeostasis and skeletal health maintenance. The presence of vitamin D receptors (VDR) in other tissue and organs suggest that vitamin D physiology extends well above and beyond bone homeostasis [1]. Additionally, the enzyme responsible for the conversion of 25[OH] D to its biologically active form [Vitamin D (1,25[OH]_2_ D)] has been identified in other tissues aside from kidneys [2,3], and that extra renal synthesis of 1,23[OH]_2_D may be equally important in regulating cell growth and differentiation via paracrine or autocrine regulatory mechanisms [4].

The mechanism of action of vitamin D_3_ through its hormonal form, dihydroxyvitaminD_3_, involves a nuclear VDR that regulates the transcription of several target genes in a variety of vitamin D target cells that are primarily involved in the calcium homeostasis of cell differentiation [5]. Hypervitaminosis D occurs when pharmacologic doses of vitamin D are consumed for prolonged periods of time or from a single megadose translating to a large increase in circulating 25[OH]D concentrations [6].

## 2. Vitamin D Metabolism

Vitamin D has two distinct forms: vitamins D_2_ and D_3_. Vitamin D_2_ is a 28-carbon molecule derived from ergosterol (a component of fungal cell membranes), while vitamin D_3_ is a 27-carbon derived from cholesterol [7]. UV-B irradiation of skin triggers photolysis of 7-dehydrocholesterol (pro-vitamin D_3_) to pre-vitamin D_3_, which is rapidly converted to vitamin D_3_ by the skin’s temperature. Vitamin D (D_2_ and D_3_) from the skin and diet undergo two sequential hydroxylations: first in the liver (25[OH]D) and then in the kidney, leading to its biologically active form 1,25-dihydroxyvitamin D (1,25[OH]_2_D) [8]. Table 1 shows the nomenclature for vitamin D precursors and metabolites.

**Table 1:** Nomenclature of vitamin D precursors and metabolites.

Common Name	Clinical Name	Abbreviation	Comments
7-Dehydrocholesterol	Pro-vitamin D_3_	7DHC	Lipid in cell membranes
Cholecalciferol	Pre-vitamin D_3_		Photosynthesized in skin or diet
Ergocalciferol	Pre-vitamin D_2_		Obtained from diet. Equivalent to vitamin D_3_ as precursor for active vitamin D
Calcidiol	25-Hydroxyvitamin D	25[OH]D	Best reflects vitamin D status
Calcitriol	1,25-Dihydroxvitamin D	1,25[OH]D_2_	Active form of vitamin D, tightly regulated

The 1,25 [OH]_2_D ligand binds with high affinity to vitamin D receptors (VDRs), which then increases intestinal absorption of both calcium and phosphorus. In addition, vitamin D is actively involved in bone formation, resorption, mineralization, and in maintenance of neuromuscular function. Circulating 1,25[OH]_2_D inhibits serum parathyroid hormone (PTH) levels by negative feedback mechanism and by increased serum calcium levels. It also regulates bone metabolism through activation of the VDRs found in osteoblasts, releasing biochemical signals and leading to the formation of mature osteoclasts [9].

In a low vitamin D state, the small intestine can absorb approximately 10%–15% of dietary calcium. When adequate however, intestinal absorption of dietary calcium rises to approximately 30%–40% [9,10]. Hence, low vitamin D levels (25[OH]D) may lead to insufficient calcium absorption, and this has clinical implications not only for bone health but also for most metabolic functions. The increase in PTH restores calcium homeostasis by increasing tubular reabsorption of calcium in the kidney, increasing bone calcium mobilization and enhancing 1,25[OH]_2_D production [10].

## 3. Optimum 25[OH]D Levels

The vitamin D level needed to optimize intestinal calcium absorption (34 ng/mL) is lower than the level needed for neuromuscular performance (38 ng/mL) [11,12]. Experts however believe that the lower limit of adequate 25[OH]D levels should be 30 ng/mL [13]. Still others recommend a lower limit of 40 ng/mL, since impaired calcium metabolism due to low serum 25[OH]D levels may trigger secondary hyperparathyroidism, increased bone turnover and progressive bone loss [14,15].

The proposed 25[OH]D cut-off for optimum skeletal health is the level that reduces PTH to a minimum and increases calcium absorption to its maximum [11,16]. Several studies have shown that PTH levels plateau at a minimum steady-state level as serum 25[OH]D levels approach and rise above approximately 30 ng/mL (75 nmol/L) [16,17,18]. The established consensus of several vitamin D cut-offs is presented in Table 2 [18,19,20]. It is noteworthy, however, that there is a continued debate and exchange of knowledge with respect to the optimum cut-off for 25(OH)D.

**Table 2:** Diagnostic Cut-Offs of levels of serum 25[OH]D.

25[OH] Level (ng/mL)	25[OH]D Level (nmoL/L)	Laboratory Diagnosis
<20	<50	Deficiency
20–32	50–80	Insufficiency
54–90	135–225	Normal in sunny countries
>100	>250	Excess
>150	>325	Intoxication

## 4. Measurements of 25[OH]D *versus* 1,25[OH]_2_D_3_

The clinical advantages of choosing 25[OH]D instead of calcitriol as a marker for vitamin D status has been listed by Rajasree *et al*. [21]. First, 25[OH]D has the highest concentration of all vitamin D metabolites. Second, its levels remain stable for almost two weeks. Lastly, vitamin D toxicity is thought to be a function of 25[OH]D instead of calcitriol. It has been observed that serum 25[OH]D is the best indicator of vitamin D status among individuals without kidney disease [22]. Furthermore, 25[OH]D in large amounts can replace calcitriol to stimulate bone calcium metabolism [23]. Although nephrectomy abolishes a response to physiological dose of 25[OH]D, a large dose (1000 fold) of 25[OH]D can stimulate intestinal calcium absorption and bone calcium metabolism in nephrectomized rats [24]. Hughes *et al.*, studied vitamin D intoxication in two human patients with normal kidney function and showed that both patients had 16-fold above normal concentrations of plasma 25[OH]D levels (500–600 ng/mL), while 1,25[OH]D_2_D_3_ plasma concentrations were only modestly elevated (40–56 pg/mL) [25]. Differences in calcidiol *versus* calcitriol are presented in Table 3.

**Table 3:** Calcidiol *versus* Calcitriol.

Metabolite function	25[OH]D	1,25[OH]_2_D_3_
Nutritional Status	Best indicator	Does not indicate nutritional status
Half life	>15 days	<15 h
Stability in serum	Stable	Unstable
Hypovitaminosis D	Indicative (low)	Non-indicative (normal to elevated)
Hypervitaminosis D	Indicative (elevated)	Non-indicative (low to normal or mild elevated)
Calcium regulation	Possible under non-physiological conditions	Tight under physiological conditions
PTH regulation	Depends on vitamin D status	Tight
DBP binding	High affinity (releases the free metabolite once DBP is saturated	Low affinity to exert the physiological function
VDR binding	Strongest among metabolite other than calcitriol	High affinity to elicit the biological function

**Note**: VDR: vitamin D receptor; DBP: vitamin D binding protein; PTH: parathyroid hormone.

## 5. Supplementation of Vitamin D_2_ *versus* Vitamin D_3_

Multiple preparations of vitamin D and its metabolites are commercially available for supplement use. The two most common supplements are ergocalciferol (vitamin D_2_) and cholecalciferol (vitamin D_3_). Some studies [26,27], but not all [28], suggest that vitamin D_3_ increases serum 25[OH]D more efficiently than vitamin D_2_. A large, single dose of vitamin D_2_ does not last longer than a large dose of D_3_. In a study conducted by Armas *et al*., [27], subjects were given one dose of 50,000 IU of either vitamin D_2_ or vitamin D_3_. Vitamin D_2_ was absorbed just as well as vitamin D_3_, yet blood levels of 25[OH]D started dropping rapidly after 3 days among subjects given vitamin D_2_ whereas those on vitamin D_3_ sustained high levels for two weeks before dropping gradually.

A daily dose of 4000 IU of vitamin D_3_ for two weeks was observed to be 1.7 times more effective in raising 25[OH]D levels than 4000 IU of vitamin D_2_ [26]. On the other hand, Holick *et al.* found that a daily dose of 1000 IU of vitamin D_2_ over 11 weeks duration increased 25[OH]D levels from 42 to 67 nmoL/L (16.9 to 26.8 ng/mL) [28]. Consequently, vitamin D_3_ levels also increased from 49 to 72 nmoL/L (19.6 to 28.9 ng/mL). It took 6 weeks for 25[OH]D levels to plateau on that regimen. In another study, Glendenning *et al.* compared 1000 IU of D_2_ *versus* D_3_ in patients who had vitamin D insufficiency with subsequent hip fractures. After three months, those who were supplemented with D_3_ had a 31%–52% greater increase in 25[OH]D levels than those supplemented with D_2_. However, parathyroid hormone levels did not differ between groups [29].

In children, Gordon *et al.*, assigned 40 infants and toddlers with vitamin D deficiency to one of three regimens (2000 IU oral vitamin D_2_ daily, 50,000 IU vitamin D_2_ weekly or 2000 IU vitamin D_3_ daily) for 6 weeks. At the end of the trial, 25[OH]D levels increased from 42.5 to 90 nmoL/L and there were no significant differences between treatment groups [30].

In terms of bioavailability, Biancuzzo *et al.*, tested changes in 25[OH]D status from a daily dose of 1000 IU of vitamin D_2_ or D_3_ from either calcium-fortified orange juice with vitamin D or supplement capsules for 11 weeks. The average 25[OH]D levels of all groups (D_2_ from orange juice, D_2_ from capsules, D_3_ from orange juice, D_3_ from capsules) went up to about 25 nmoL/L with no significant differences between groups [31].

Treatment for most studies found D_2_ to be less effective than D_3_, whereas in studies finding them equally effective, the treatment was daily amounts between 400 and 2000 IU [32]. Houghton and Vieth indicated that vitamin D_3_ is the most potent form of vitamin D in all primate species, including humans, owing to the diminished binding of vitamin D_2_ metabolites to DBP in plasma [33]. They also confirmed the finding of Hollick [34], which indicated that the difference in binding capacity is potentially explained by the presence of a methyl group at carbon-24 position on the D_2_ molecule. The different hydroxylation sites of two forms of vitamin D leads to the production of unique biologically active metabolites. Based on this, the 24-hydroxylation after the 25-hydroxylation results in the formation of 1,24,25[OH]_3_D_2_ and the deactivation of vitamin D_2_ molecule. On the other hand, the vitamin D_3_ metabolite 1,24,25[OH]_3_D_3_ must undergo an additional side chain oxidation to be biologically deactivated [35]. Interestingly, 1,24,25[OH]_3_D_3_ has the ability to bind VDR with ~40% capacity higher than with 1,25[OH]_2_D_3_ [36].

## 6. Candidates for Calcidiol (25-OHD) Measurements

The best indicator of vitamin D status is 25-OHD because it reflects cutaneous and dietary intake, not to mention it is the major circulating form of vitamin D [37]. While there are many established causes of vitamin D deficiency, as listed in Table 4, screening for the general population warrants further investigation. The United States Preventive Services Task Force (USPSTF) did not comment for or against routine screening for vitamin D deficiency. One approach is to consider serum testing in patients at high risk for vitamin D deficiency, and treating without testing those at a lower risk [38]. Just recently, a statement from Osteoporosis Canada suggested that based on clinical suspicion for vitamin D insufficiency and its complications the clinical approach can take into account three settings (Table 5).

**Table 4:** Major causes of vitamin D deficiency [13].

Causes	Example
Reduced skin synthesis	Sunscreen, skin pigment, season/time of day, aging
Decreased absorption	Cystic fibrosis, celiac disease, Crohn’s disease, gastric bypass, medications that reduce cholesterol absorption
Increased sequestration	Obesity (BMI > 30)
Increased catabolism	Anti-convulsant, glucocorticoid
Breastfeeding	Exclusively without vitamin D supplementation
Decreased synthesis of 25-hydroxyvitamin D	Hepatic failure
Increased urinary loss of 25-hydroxyvitmain D	Nephrotic proteinuria
Decreased synthesis of 1,25-dihydroxyvitmain D	Chronic renal failure
Inherited disorders	Vitamin D resistance

**Table 5:** Approach to vitamin D correction [39].

Risk Category	Action	Level of Evidence
Low: Adult < 50 years Without comorbid conditions affecting vitamin D absorption or action	400–1000 IU No calcidiol measurement required	Level 3 Evidence grade D
Moderate: Adult > 50 years With or without osteoporosis but without comorbid conditions that affect vitamin D absorption or action	800–2000 IU Calcidiol measurement in initial assessment but if therapy for osteoporosis is prescribed, calcidiol should be measured after three to four months, of an adequate dose.	Level 2 Evidence grade B Level 3 Evidence grade D
High: Co-morbid conditions that affect vitamin D absorption or action and/or recurrent fractures or bone loss despite osteoporosis treatment	Calcidiol should be measured and supplementation based on the measured value.	Grade B Recommendation

## 7. Vitamin D Correction

In patients with normal absorptive capacity, for every 40 IU/day (1 µg/day) of vitamin D_3_, serum 25(OH)D concentrations increase by approximately 0.3 to 0.4 ng/mL (0.7 to 1.0 nmol/L) [40]. Largest increments are seen in patients with the lowest starting 25(OH)D level, but subsequently declines as 25(OH)D concentration reaches 40 ng/mL (100 nmol/L) [41]. Nutritional deficiency (25OHD < 50 nmol/L) requires initial treatment with 50,000 units of vitamin D_2_ or vitamin D_3_ orally once per week for 6–8 weeks, and then 800 to 1000 IU of vitamin D_3_ orally thereafter [42]. Intramuscular cholecalciferol (300,000 IU) in one or two doses per year is also an option for increasing serum 25 OHD level [43].

Nutritional insufficiency (25 OHD 50–75 nmol/L) requires treatment with 800 to 1000 IU of vitamin D_3_ daily. This intake will bring the average adult’s vitamin D status to 7 nmol/L higher over a three-month period. Still, many individuals might need higher doses. In malabsorptive states, oral dosing and duration of treatment is dependent on the individual patient’s on vitamin D absorptive capacity. High doses of vitamin D (10,000 to 50,000 IU daily) may be necessary for patients who had gastrectomy or malabsorption history. Patients who remain deficient or insufficient on such doses need to be treated with hydroxylated vitamin D metabolites, since they are more readily absorbed than with ordinary sun or sun camp exposure. All patients should maintain a daily calcium intake of at least 1000 mg (for ages 31 to 50 years) to 1200 mg (for ages 51 and older) per day [44].

## 8. Vitamin D Toxicity

Vitamin D as a fat-soluble vitamin raised concerns about toxicity from excessive supplementation. Widespread vitamin D fortification of foods and drinks from the 1930s to 1950s in the United States and Europe led to reported cases of toxicity [45]. Hypercalcemia is responsible for producing most of the symptoms of vitamin D toxicity. Early symptoms of vitamin D toxicity include gastrointestinal disorders like anorexia, diarrhea, constipation, nausea, and vomiting. Bone pain, drowsiness, continuous headaches, irregular heartbeat, loss of appetite, muscle and joint pain are other symptoms that are likely to appear within a few days or weeks; frequent urination, especially at night, excessive thirst, weakness, nervousness and itching; kidney stones [46].

There are three major hypotheses for vitamin D toxicity [47]:

(i) **Raised plasma 1,25[OH]D concentrations lead to increased intracellular 1,24[OH]D concentrations.** This hypothesis is not widely supported as many studies revealed that vitamin D toxicity is associated with normal or marginally elevated 1,25[OH]D [23]. It was only Mawer *et al.* who reported elevated 1,25[OH]D with vitamin D toxicity [48].
(ii) **Vitamin D intake raises plasma 25[OH]D levels to concentrations that exceed DBP binding capacity, and free 25[OH]D has direct effects on gene expression once it enters target cells.** High dietary vitamin D intake alone increases plasma 25[OH]D. The low affinity of 1,25[OH]D for the transport protein DBP and its high affinity for VDR dominate normal physiology. This makes it the only ligand with access to the transcriptional signal transduction machinery. However, in vitamin D intoxication, overloading by various vitamin D metabolites significantly compromises the capacity of the DBP by allowing other metabolites to enter the cell nucleus. Of all the inactive metabolites, 25[OH]D has the strongest affinity for the VDR, and thus at sufficiently high concentrations, could stimulate transcription [47].
(iii) **Vitamin D intake raises the concentrations of many vitamin D metabolites**, including vitamin D itself and 25[OH]D, and these concentrations exceed the DBP binding capacity and release of “free” 1,25[OH]D which enters target cells [47].

The amount of UVB radiation required for vitamin D sufficiency can be calculated from the amount of vitamin D produced from one minimal erythemal dose (MED), or 10,000–25,000 IU of oral vitamin D [9].The MED can be defined as the amount of time needed to cause skin to turn pink. The length of time varies with geographical location, skin pigmentation, percent of body fat, and age. Excessive exposure to sunlight will not cause vitamin D intoxication because sunlight degrades any excess vitamin D [48].

The highest recorded individual serum 25[OH]D concentration obtained from sunshine was from a farmer in Puerto Rico with a level of 225 nmol/L [49]. On the other hand, the highest recorded individual 25[OH]D achieved from artificial ultraviolet light treatment sessions was 275 nmol/L [50]. Vieth reported that vitamin D toxicity probably begins to occur after chronic consumption of approximately 40,000 IU/day (100 of the 400 IU capsules) [6]. Reports in which pharmacologic doses of vitamin D were given for a prolonged time, the indications why it was given and in which the final serum 25[OH]D concentrations are provided and summarized in Table 6.

## 9. Hypersensitivity to Vitamin D

Vitamin D hypersensitivity syndromes are often mistaken for vitamin D toxicity. The most common is primary hyperparathyroidism. Granulomatous diseases, such as sarcoidosis, granulomatous TB and some cancers also cause vitamin D hypersensitivity, as the granuloma or the tumor may make excessive amounts of calcitriol, thus raising serum calcium levels [6].

**Table 6:** Studies reporting elevated vitamin D status and associated diseases.

Reference, year, and daily dosage (µg)	Duration	Final 25[OH]D concentration (nmoL/L)	Indication
Mason *et al.*, [51], 1980 1250	>52 weeks	717	Hypoparathyroidism
Haddock *et al.*, [49], 1982 1875	>100 weeks	1707.5	Hypoparathyroidism
Gertner and Domenech [52], 1977 500–2000	12–52 weeks	442–1022	Various
Counts *et al.*, [53], 1975 2500	12 weeks	1550	Anephric
Hughes *et al.*, [25], 1976 2500–6250 *n* = 3	>52 weeks	1000–1600	Not stated
Streck *et al.*, [54], 1979 2500	3.8 years	707.5	Hypoparathyroidism
Davies and Adams [55], 1978			
3750	364 weeks	1125	Paget disease
2500	520 weeks	1000	Thyroidectomy
Mawer *et al.*, [48], 1985			Hypoparathyroidism
1875	520 weeks	568	Hypophosphatemic
5000	520 weeks	1720	rickets
2500	520 weeks	995	Carpal tunnel
1250	1248 weeks	632	syndrome
4285	26 weeks	908	Celiac disease
2500	520 weeks	856	Chilblain
2500	312 weeks	778	Thyroidectomy
1250	1040 weeks	903	Arthritis
			Hypoparathyroidism
Allen and Skah [56], 1992			Hypoparathyroidism
1875	19 years	267	
Rizzoli *et al.*, [57], 1994			
15,000	96 weeks	221	
7500	3 weeks	801	Osteoporosis
7500	74 weeks	1692	Osteoporosis
1075	12 weeks	374	Hypoparathyroidism
7500	4 weeks	650	Osteoporosis
7500	4 weeks	621	Osteoporosis
250	390 weeks	608	Osteomalacia
Pettifor *et al.*, [58] 1995			Not stated
50,000 (*n* = 11)	10 days	847–1652	
Jacobus *et al.*, [59] 1992			Not stated
725–4364 (*n* = 8)	6 years	“mean” 731	

## 10. Conclusions

The present review discussed current knowledge on vitamin D physiology, its clinical relevance and evidence-based treatment options on vitamin D status correction. Caution should still be practiced by clinicians in providing vitamin D supplementation among vitamin D deficient populations, with proper monitoring using approved and certified methods. Indications for vitamin D supplementation outside the conventional calcium homeostasis should also be considered to maximize extra-skeletal benefits of vitamin D correction.
